# Enterovirus D68 Infection

**DOI:** 10.3390/v7112925

**Published:** 2015-11-24

**Authors:** Susanna Esposito, Samantha Bosis, Hubert Niesters, Nicola Principi

**Affiliations:** 1 Pediatric Highly Intensive Care Unit, Department of Pathophysiology and Transplantation, Università degli Studi di Milano, Fondazione IRCCS Ca’ Granda Ospedale Maggiore Policlinico, 20122 Milan, Italy; samantha.bosis@unimi.it (S.B.); nicola.principi@unimi.it (N.P.); 2 Department of Medical Microbiology, Division of Clinical Virology, University Medical Center, The University of Groningen, 9713 Groningen, The Netherlands; maria.favero@policlinico.mi.it

**Keywords:** acute flaccid paralysis, children, Enterovirus D68, EV-D68, respiratory tract infections

## Abstract

First described in 1962 in children hospitalized for pneumonia and bronchiolitis, the Enterovirus D68 (EV-D68) is an emergent viral pathogen. Since its discovery, during the long period of surveillance up to 2005, EV-D68 was reported only as a cause of sporadic outbreaks. In recent years, many reports from different countries have described an increasing number of patients with respiratory diseases due to EV-D68 associated with relevant clinical severity. In particular, an unexpectedly high number of children have been hospitalized for severe respiratory disease due to EV-D68, requiring intensive care such as intubation and mechanical ventilation. Moreover, EV-D68 has been associated with acute flaccid paralysis and cranial nerve dysfunction in children, which has caused concerns in the community. As no specific antiviral therapy is available, treatment is mainly supportive. Moreover, because no vaccines are available, conventional infection control measures (*i.e.*, standard, for contacts and droplets) in both community and healthcare settings are recommended. However, further studies are required to fully understand the real importance of this virus. Prompt diagnosis and continued surveillance of EV-D68 infections are essential to managing and preventing new outbreaks. Moreover, if the association between EV-D68 and severe diseases will be confirmed, the development of adequate preventive and therapeutic approaches are a priority.

## 1. Introduction

First described in 1962 by Schieble *et al.* in four Californian children hospitalized for community-acquired pneumonia and bronchiolitis, Enterovirus D68 (EV-D68) is an emergent viral pathogen belonging to the species *Enterovirus D*, genus *Enterovirus*, family Picornaviridae, and order Picornavirales [1,2].

EV typing is based on comparing the sequences encoding the VP1 capsid protein: viruses of different genotypes have <75% nucleotide identity and <85% amino acid identity [3,4]. EV-D68 is a non-enveloped, single-stranded RNA virus composed of four structural proteins: VP1, VP2, VP3, and VP4. On the basis of the VP1 nucleotide sequence, EV-D68 strains isolated in recent years have been classified into three genetic groups named differently by the authors (*i.e.*, lineages 1, 2 and 3 or clades A, B, and C) [5]. EV-D68 is a particular EV. It shares similar biological characteristics with rhinoviruses (RVs), including acid sensitivity and growth at lower optimal temperature than other EVs [6]. Moreover, to enter the respiratory cells, it binds specifically to α2-6 sialic acid instead of to α2-3 sialic acid, which is the receptor of the other EVs [7]. Finally, it can be neutralized by a monotypic antiserum specific for RV87, suggesting that these two viruses have significant genetic similarities and are strains of the same picornavirus serotype that presents features of both RVs and EVs [3].

After the initial detection, the largest and most widespread EV-D68 outbreaks were observed in August 2014 when authors initially from the United States and then from different countries reported an increased number of respiratory diseases due to EV-D68 [1,2,3,4,5,6,7,8,9,10,11,12,13,14,15,16,17,18,19,20,21,22,23,24]. The unexpectedly high number of children hospitalized with severe respiratory illness and neurological complications due to EV-D68 infection have raised concerns in the international community [1,22,25,26,27,28,29]. This review summarizes the current knowledge regarding the epidemiological, pathogenic, and clinical characteristics of EV-D68 infection. 

## 2. Epidemiology

Although few EV-D68 cases were detected until 2005, sporadic outbreaks of EV-D68 respiratory disease were nonetheless reported from the United States, The Netherlands, the Philippines, and Japan [30]. The circulation of different EV-D68 strains has been described worldwide during the last five years. In 2014, the United States and Canada had the largest and most widespread EV-D68 outbreaks, and these were associated with severe clinical manifestations [1,25,26,27,28,29]. Subsequent reports have documented outbreaks of respiratory diseases caused by EV-D68 in Europe, Thailand, China, New Zealand, and several African countries [1,5,8,9,10,11,12,13,14,15,16,17,18,19,20,21,22,23,24], and these data have demonstrated an increasing number of patients with EV-D68 infections in recent years. Initially, the increase was in large part ascribed to the introduction into clinical practice of new methods for identifying infectious agents, mainly based on molecular biology, which were significantly more sensitive than the traditional culture previously used for viral detection. However, retrospective studies carried out with molecular methods on stored respiratory samples showed that the incidence of EV-D68 had actually increased [31].

Meijer *et al.* [32] retrospectively tested specimens collected from acute respiratory infections surveillance and through three children cohort studies conducted in The Netherlands from 1994 through 2010. A total of 71 of 13,310 (0.5%) specimens were positive for EV-D68, with an almost annual circulation of the virus.

The median age of 19 patients with EV-D68 identified in Kansas City during the 2014 USA outbreak was four years, with an age range of six weeks to 16 years [1,9]. Similar data were collected in Chicago during the same outbreak, with the median age of the 11 patients with EV-D68 being five years, ranging from 20 months to 15 years [1,9]. Finally, among the 389 patients with EV-D68 identified in Europe, there were 120, 135, 56, and 78 aged 0–1, 2–5, 6–16, and ≥17 years, respectively [33]. However, because most EV-D68 studies to date have targeted paediatric patients, the true susceptibility to EV-D68 of different age groups has not been fully defined. Indeed, it cannot be excluded that the incidence of EV-D68 in adults is significantly higher than generally thought [10,32,33,34].

The phylogenetic analysis of EV-D68 sequences showed that three different distinct viral clades (genetically similar groups that share a common origin) have emerged and are currently circulating worldwide. Since the 1960s, the EV-D68 genome showed an increased VP1 gene diversity that resulted in a division in clades A, B, and C [35].

In the majority of EV-D68 cases reported worldwide, children represent the main population affected because these were the patients who were preferentially sampled and tested. Moreover, unlike other enteroviruses, EV-D68 has a seasonal pattern with a high incidence in early autumn and winter in temperate climates [1,5].

## 3. Biological Characteristics and Pathogenesis

The EV genome consists of an RNA strand of approximately 7500 nucleotides and contains a single open reading frame (ORF) encoding a polyprotein flanked by untranslated regions (UTRs) at each end; the 5′ UTR contains the highly conserved internal ribosome entry site (IRES) [1]. The VP1 gene, which encodes one of four capsid proteins, has traditionally been used to distinguish between enteroviral serotypes. In the past, *Enterovirus* taxonomy included polioviruses, coxsackie viruses A and B, and EVs, although the taxonomy has recently changed and now includes four human species based on molecular and biological characteristics (*i.e.*, enteroviruses A, B, C, and D), human RVs and some animal EVs [3]. Each species includes different serotypes [1,35].

Among EV D isolates, five types have been identified, and four (EV-D68, EV-D70, EV-D94, EV-D111) have been found to infect humans. EV-D68 is considered an atypical EV, sharing some biological characteristics with RVs. Unlike the majority of EVs, EV-D68 grows at lower temperatures (at 33 °C instead of 37 °C) and does not tolerate acidic conditions [1]. These characteristics could most likely explain the predilection of EV-D68 for the respiratory rather than the gastrointestinal tract, making this virus more similar to RVs than EVs. For all these reasons, EV-D68 was initially named human RV87 until genetic and antigenic analysis led to its re-classification as an enterovirus [1].

Different from other EVs, which are spread mainly via the faecal-oral route, EV-D68 can be transmitted through the inhalation of aerosolized particles or the manual transfer of infected material to the airway from environmental surfaces [3,33]. After attachment to the epithelial cells of the mucosa of the upper respiratory tract, EV-D68 spreads locally and then throughout the organism [1]. EV-D68 has been detected in the blood of few patients [35] and the neurologic involvement due to EV-D68 is consistent with tropism of this virus for motor nerve cells [3].

Because the temperature in the nose is lower than in the lungs and α2-6 sialic acid receptors are dominantly expressed in the upper respiratory tract, EV-D68 should predominantly cause upper respiratory tract infections (URTIs). Regardless, several epidemiological studies have shown that during EV-D68 epidemics, lower respiratory tract infections (LRTIs) are very common and, in several instances, more common than URTIs. Although this finding could be caused by a sampling bias and it is not clear whether EV-D68 is truly causing LRTI (*i.e.*, pneumonia) or it is causing severe reactive airway disease (*i.e.*, wheezing and asthma exacerbations), this suggests that EV-D68 uses alternative mechanisms to enter cells, permitting invasion and damage of the lower respiratory tract. Because EV-D70, a virus belonging to the same genetic cluster as EV-D68, uses decay-accelerating factor (DAF) for cell entry and because this factor is expressed at high levels in epithelial cells of the lower respiratory tract, a potential hypothesis could be that this mediator plays a significant role in explaining the higher-than-expected risk of LRTIs in cases of EV-D68 [36].

There are at least three major clades of EV-D68 currently in worldwide circulation, A, B, and C, which most likely originate from diversification of the viral genome [35]. Moreover, within the clades, subgroups are identified according to amino acid substitutions in both structural and nonstructural proteins. Since the 1960s, the EV-D68 genome has undergone many deletions in the spacer region of the 5′ UTR between the end of the IRES and the polyprotein ORF. It is believed that these deletions might affect the virulence of the virus by enhancing translational efficiency, which might be related with the recent increase in EV-D68 cases worldwide [35]. During the 2014 USA outbreak, seven strains of EV-D68 included in clade B were identified [35], and strains with 95% identity were identified in The Netherlands, Italy, and China in the same period [33,35].

## 4. Clinical Features

EV-D68 has been reported to cause mild to severe upper and lower respiratory illnesses. In sentinel general practice network surveillance for influenza illness conducted in The Netherlands, the EV-D68 detection rate was found to be higher among patients than controls, demonstrating the role of this virus in respiratory diseases [32].

EV-D68 infection is characterized by sudden onset of the disease, and the major respiratory symptoms include fever, rhinorrhoea, sneezing, malaise, cough, and sore throat [8,15,31]; pneumonia and asthma exacerbation represent the main complications due to EV-D68 [15,16]. The results from national EV-D68 surveillance in The Netherlands from 2011 and 2014 showed that outpatients presented with mild upper or lower respiratory infections, with the main symptoms being cough and fever; however, hospitalized patients had more severe respiratory diseases [10]. In an epidemiological study in the Philippines, the EV-D68 detection rate was higher among children hospitalized with pneumonia than among outpatients with other respiratory infections [37]. Poelman *et al.* [17] analysed 1896 respiratory samples collected during the first three quarters of 2014 in The Netherlands and found 18 cases positive for EV-D68, 11 of which were children younger than 18 years old. Five children presented with severe respiratory illness and were hospitalized in the paediatric intensive care unit (PICU), requiring intubation and mechanical ventilation [17].

During the recent outbreaks in Kansas City and Chicago in 2014, many of the hospitalized children had clinical manifestations of severe illness, including difficulty in breathing, hypoxaemia and respiratory distress, largely without fever [1,9]. Chest radiographs were normal or showed hyperinflation or perihilar infiltrates. Most of the children were admitted to the PICU and required mechanical ventilation or bi-level positive airway pressure ventilation [1,9].

The overall data from different countries suggest that EV-D68 infections are more likely to be associated with severe and life-threating respiratory diseases than that caused by other EVs. Many EV-D68 infections appear to be more severe in patients with underlying conditions such as prematurity or chronic diseases, in immunocompromised children and in those with a history of asthma or wheezing [9,10,16,17,18,19,20,31,38]. Moreover, an epidemiological study from Denmark that screened 130 respiratory samples from both children and adults for EV-D68 showed that patients with underlying conditions had a longer duration of illness (more than three weeks) compared with healthy subjects (from 4 to 14 days) [18].

A recent prospective study investigated the potential involvement of EV-D68 in determining community-acquired pneumonia (CAP) in children hospitalized between June and December 2014, the same period during which outbreaks of EV-D68 infection were reported in the United States. The authors reported 97 (55.1%) patients with nasopharyngeal samples positive for EVs or RVs, and of these, four (2.3%) were positive for EV-D68 [20]. The chest radiographies of all EV-D68-positive patients showed alveolar CAP; three patients had a mild-to-moderate respiratory illness, and their clinical outcome was favourable. The most severe case was a girl aged 14 years old with mitochondrial encephalopathy, lactic acidosis and stroke-like episodes (MELAS syndrome) and an infection due to *Pseudomonas aeruginosa* who died [20]. Although EV-D68 appears to primarily cause respiratory illness, the full spectrum of diseases remains unclear.

Some reports have even associated this virus with central nervous system diseases. Many cases of neurological complications associated with respiratory illnesses in children have been reported in the United States and Canada [9,39]. EV-D68 has been detected in the cerebrospinal fluid (CSF) in one case of a 5-year old boy who died due to meningoencefalitis and pneumonia [40]. Massacar *et al.* [29] reported a geographically and temporally defined cluster of acute flaccid paralysis and cranial nerve dysfunction in children associated with an outbreak of EV-D68 respiratory illness in Colorado in 2014. The authors demonstrated that during the study period, there was a 36% increase in respiratory visits at the emergency department and a 77% increase in admissions for respiratory symptoms compared to the corresponding month in the years prior. EV-D68 was identified in 19 of 25 patients (76%) hospitalized in the PICU with severe respiratory diseases. Moreover, 12 patients (median age 11.5 years) had acute flaccid paralysis and/or cranial nerve dysfunctions. All of these children exhibited a febrile illness associated with respiratory symptoms before the onset of neurological disease. Although EV-D68 was not detected in the CSF from any of these patients, but only in nasopharyngeal samples from 5 (45%) of 11 patients, these data appear to suggest the role of this virus in the pathogenesis of neurological diseases [29].

Recently, cases of acute flaccid paralysis have also been described in Europe. Lang *et al.* [27] reported the case of a previously healthy four-year-old boy in France hospitalized with meningeal syndrome and pneumonia in September 2014. After a few days, the patient was transferred to the PICU for acute respiratory distress, after which he developed flaccid tetraparalysis. In this case, EV-D68 was isolated from stool, nasopharyngeal aspirates and broncho-alveolar lavage, showing a possible link between this virus and the severe neurological complications. In Norway, two cases of severe acute flaccid myelitis occurred in two children aged five and six years old in autumn 2014 [26]. Both children had febrile respiratory illness prior to the development of neurological symptoms, and EV-D68 was isolated only from respiratory samples.

Regarding neurological images, some authors have reported that patients presenting with acute flaccid paralysis and/or cranial nerve dysfunction during outbreaks demonstrate unique imaging findings characterized by brain stem and grey matter spinal cord lesions, similar to findings described in previous outbreaks of poliomyelitis [41].

## 5. Diagnosis, Treatment and Prevention

Testing for EV-D68 is usually performed when a child has a severe respiratory illness and the cause is unknown. Virus isolation from cell culture has been used for many years for the detection of enteroviruses. However, molecular diagnostic methods such as the reverse transcriptase-polymerase chain reaction (RT-PCR) has recently replaced culturing and is actually considered the method of choice for the isolation of these viruses, particularly for examining respiratory secretions and CSF. RT-PCR is generally more sensitive and more rapid than culturing [42]. Unfortunately, not all laboratories are equipped to conduct testing for EVs. Moreover, many available laboratory methods for the detection of EVs do not distinguish between EVs and RVs and/or provide no information on serotype [31,42,43]. Some of the RVs severe infections previously described during the 2009–2014 period were EV-D68 in reality [44,45].

No specific antiviral therapy is available to treat EV-D68 infection. The Centers for Disease Control and Prevention (CDC) in the United States has tested many antiviral drugs that appeared to have activity against some EVs [1]. Among the 16 tested drugs, four (KR-22865, rupintrivir, V-7404 and DAS181) were found to have strong inhibitory activity, with a half-maximal effective concentration of 0.0012–0.0051 μM [3,46,47]. However, among these drugs, rupintrivir and KR-22865, an EV/RV protease inhibitor and an EV/RV 2C inhibitor, respectively, were abandoned; these drugs are not currently being developed. Moreover, V-7404, an EV/RV protease inhibitor, is being developed in combination with pocapavir for the treatment of poliovirus infection, and DAS 181, a sialidase fusion protein, is being developed to treat influenza and parainfluenza infections. In particular, these two last compounds are possible drug candidates for alleviating EV-D68 outbreaks. A certain degree of activity is also ascribed to pleconaril, an EV/RV capsid inhibitor, although the available data regarding its efficacy are conflicting.

In fact, treatment for EV-D68 infections is mainly supportive. Mild cases of EV-D68 infections are self-limited and require no treatment other than supportive therapies, whereas children with more severe illness require hospitalization and often intensive care interventions, including mechanical ventilation [3,31].

Because no vaccines are available for EV-D68, conventional infection control measures (*i.e.*, standard, for contacts and droplets) in both community and healthcare settings are recommended [1,3,31,42].

## 6. Conclusions

Due to the high number of cases of severe respiratory infection and the association with neurological complications, EV-D68 appears to be more virulent and dangerous for the community than previously thought. Moreover, the simultaneous circulation of similar strains of EV-D68 among countries indicates that this virus could periodically cause epidemics worldwide. However, further studies are required to fully understand the true importance of EV-D68 infections in different age groups. The prompt diagnosis and continued surveillance of EV-D68 infections is essential to manage and prevent new outbreaks. Moreover, if the association between EV-D68 and severe diseases is confirmed, the development of adequate preventive and therapeutic approaches will be a priority.
